# Supplementary material for: Environment geometry alters subiculum boundary vector cell receptive fields in adulthood and early development
Source: Nat Commun. 2024 Feb 1;15:982. doi: 10.1038/s41467-024-45098-1 (PMC10834499; doi:10.1038/s41467-024-45098-1)
Supplement: Supplementary file 1 — Supplementary Information [file 41467_2024_45098_MOESM1_ESM.pdf]

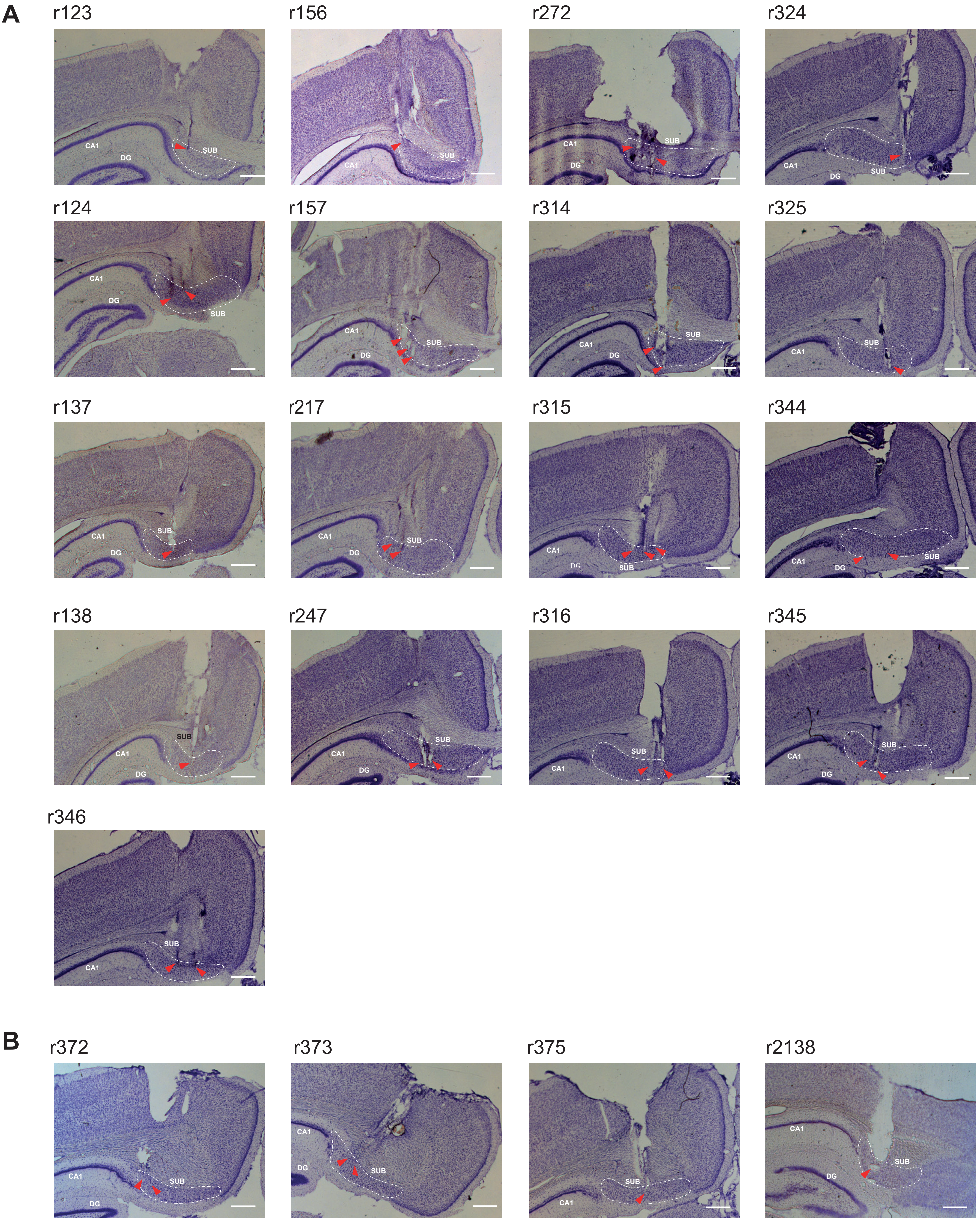

Supplementary Figure 1. Representative post-mortem histological sections (one per rat; r2138 - saggittal section, remaining brains are coronal sections), showing tetrode positions in the subiculum, for developing (**A**) and adult (**B**) subjects. White dashed boxes show the extent of the subiculum, red arrowheads indicate show the deepest extent of the tetrode track in the subiculum. CA1, Hippocampus Cornu Ammonis field 1, DG, Dentate Gyrus, Sub, Subiculum. Scale bar shows 500µm.

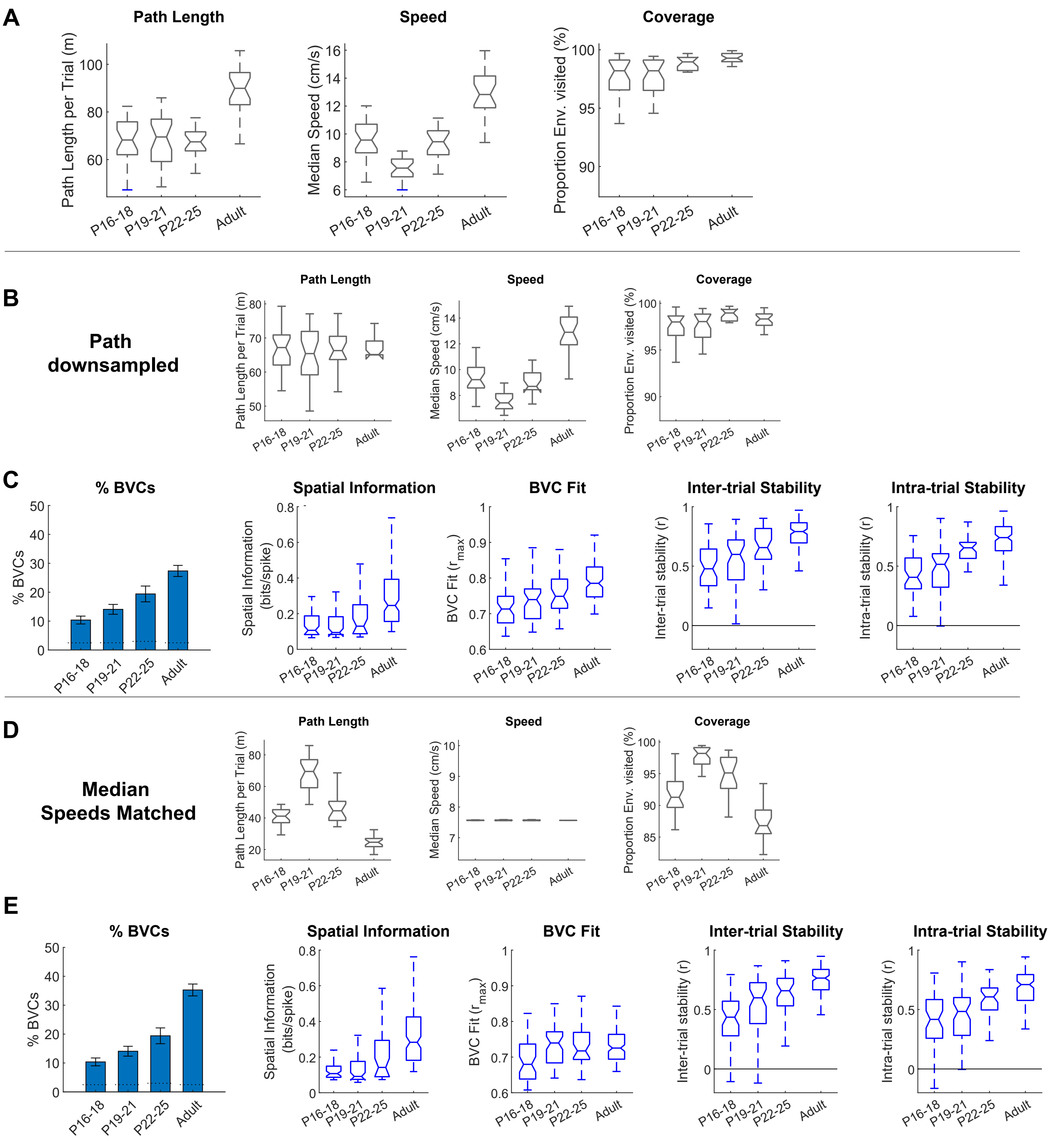

Supplementary Figure 2. The development of exploratory behaviour. Path length, running speed and arena coverage significantly change with age, but do not drive age-related changes in BVC spatial firing.

(**A**) Quantification of exploratory behaviour for all subiculum-implanted rats. Path length per trial, median running speed per trial, and the percentage coverage of the arena all significantly changed with age (ANOVA Age: Path length $F_{(3,92)}=29.2$, $p<0.001$; Speed, $F_{(3,92)}=71.3$, $p<0.001$; Coverage $F_{(3,92)}=5.27$, $p=0.002$), though running speed did not increase monotonically, but was minimal around weaning, as previously observed (see main text). n=96 trials.

(**B**,**C**) Removing positional data to equalize path lengths over age (B; Sub-sampled data ANOVA $F_{(3,92)}=0.16$, $p=0.93$) also removed the effect of age on arena coverage (ANOVA $F_{(3,92)}=1.98$, $p=0.13$), but did not affect the developmental trends reported in main figure 2 (C; ANOVA Age, Spatial Information $F_{(3,279)}=17.3$, $p<0.001$; BVC $r_{(max)}$, $F_{(3,279)}=24.4$, $p<0.001$; Inter-trial stability, $F_{(3,278)}=45.1$, $p<0.001$; Intra-trial stability $F_{(3,279)}=50.0$, $p<0.001$). n=283 BVCs.

(**D**,**E**) Sub-sampling position data to match trial median speeds across ages (D) also did not affect the developmental trends in BVC development reported in main figure 2 (E; ANOVA Age, Spatial Information $F_{(3,283)}=32.3$, $p<0.001$; BVC $r_{(max)}$, $F_{(3,283)}=8.45$, $p<0.001$; Inter-trial stability, $F_{(3,282)}=41.0$, $p<0.001$; Intra-trial stability $F_{(3,283)}=46.3$, $p<0.001$), though adult BVC $r_{(max)}$ is comparatively lowered and similar to P25 values (Tukey HSD, $p=0.89$), most likely due to the reduced arena coverage following median speed matching (D). Adult values remained nevertheless significantly greater than P16 ($p=0.002$). n=287 BVCs.

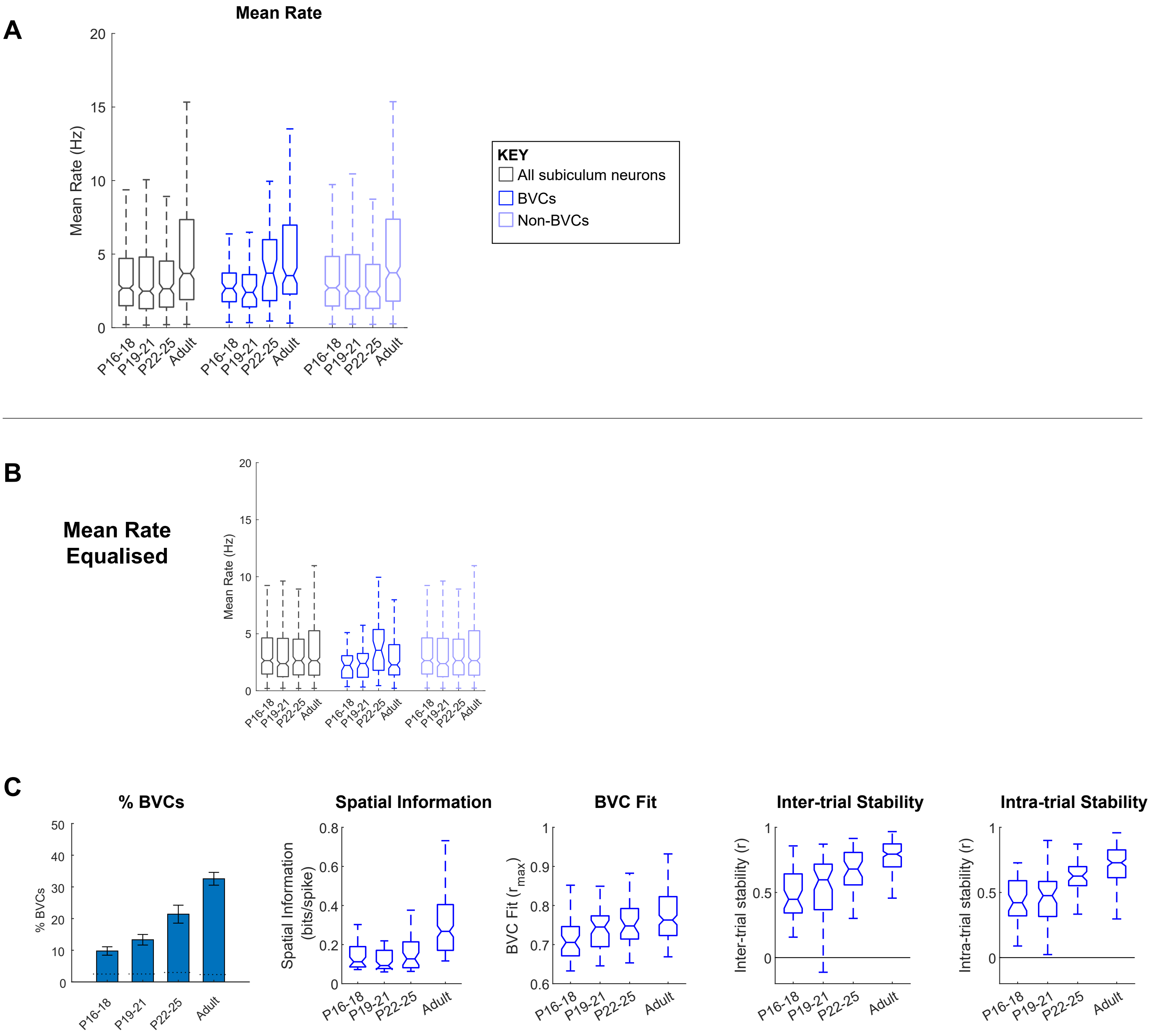

Supplementary Figure 3. Quantification of mean rate of subiculum neurons across development. Developmental increases in population mean rate do not drive age-related changes in BVC spatial firing.

(**A**) Boxplots showing mean rate of all recorded subiculum neurons, BVCs, and subiculum neurons which were not BVCs. In all three categories, mean rate significantly increases with age (ANOVA Age: All Subiculum, $F_{(1,1593)}$=20.8, p<0.001; BVCs, $F_{(1,364)}$=12.8, p<0.001; non-BVCs, $F_{(1,1225)}$=15.6, p<0.001). There is no difference between the mean rates of BVC and non-BVC neurons (ANOVA Age, Cell Type: Cell Type, $F_{(1,1597)}$=1.96, p=0.16; Age*Cell Type, $F_{(3,1597)}$=1.10, p=0.35). n=1597 subiculum neurons.

(**B**,**C**) Equalising mean rate over age by random sub-sampling of spikes does not change development trends in BVC firing, as reported in main figure 2. (**B**) After equalisation of mean rates (performed on the full subiculum population), there is no significant effect of age on the rates of all subiculum neurons (ANOVA Age: $F_{(3,1593)}$=1.35, p=0.26), though there remained an effect of age on those cells classified as BVCs ($F_{(3,316)}$=4.89, p=0.002). (**C**) In rate-equalised data, the spatial information, BVC model fit, inter- and intra-trial stability of BVCs all significantly increase with age (ANOVA Age, Spatial Information $F_{(3,307)}$=21.9, p<0.001; BVC $r_{(max)}$, $F_{(3,307)}$=13.4, p<0.001; Inter-trial stability, $F_{(3,306)}$=48.2, p<0.001; Intra-trial stability $F_{(3,307)}$=53.6, p<0.001).

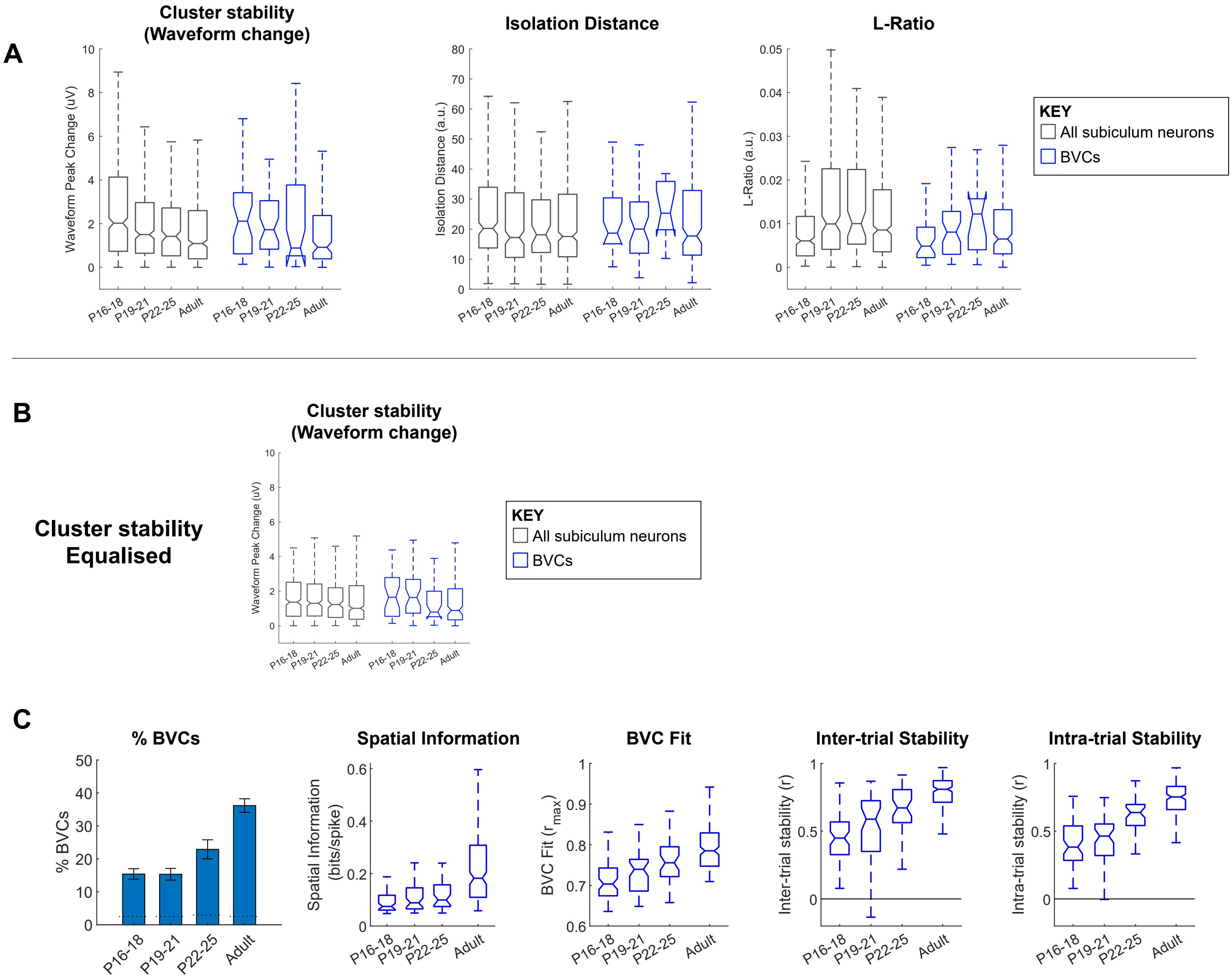

Supplementary Figure 4. Quantification of extracellular spike cluster quality and stability. Age-related changes in BVC spatial firing are not caused by changes in cluster isolation or stability.

(**A**) Boxplots showing distributions of inter-trial cluster stability (expressed as absolute change in peak waveform voltage; left) and cluster isolation quality (quantified using Isolation Distance and L-Ratio; middle and right). Cluster isolation quality does not significantly change with age, whether considering BVCs selectively (ANOVA Age: Isolation distance, $F_{(3,364)}$=0.63, p=0.59; L-Ratio, $F_{(3,364)}$=1.00, p=0.393) or considering all subiculum neurons (Isolation distance, $F_{(3,1593)}$=1.28, p=0.28; L-Ratio, $F_{(3,1593)}$=1.93, p=0.085). Stability does not change with age for BVCs (ANOVA Age, $F_{(3,364)}$=1.67, p=0.173), but does significantly decrease with age when considering all subiculum neurons ($F_{(3,1593)}$=13.7, p<0.001). n=1597 subiculum neurons.

(**B**,**C**) Equalising cluster stability across age groups, by excluding lower stability cells does not change development trends in BVC firing, as reported in main figure 2. (**B**) After equalisation, stability does not significantly change over age, for all subiculum neurons (ANOVA age, $F_{(3,1431)}$=0.11, p=0.955). (**C**) In stability-equalised data, the spatial information, BVC model fit, inter- and intra-trial stability of BVCs all significantly increase with age (ANOVA Age, Spatial Information $F_{(3,330)}$=14.4, p<0.001; BVC $r_{(max)}$, $F_{(3,330)}$=37.7, p<0.001; Inter-trial stability, $F_{(3,329)}$=71.8, p<0.001; Intra-trial stability $F_{(3,300)}$=94.6, p<0.001).

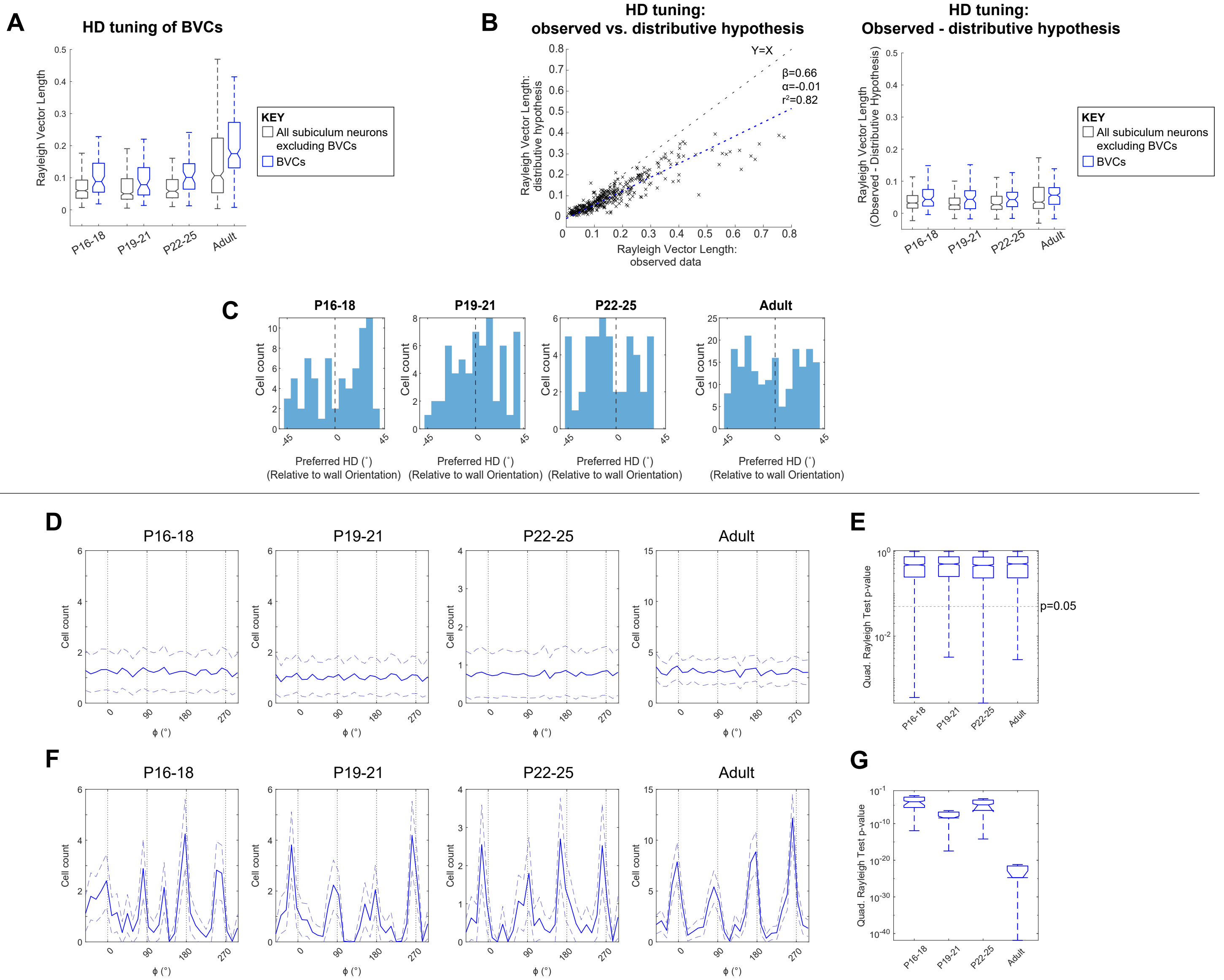

Supplemental figure 5. BVCs have low levels of head direction tuning, which is largely explained by positional sampling bias. Simulation of BVCs indicates that four-fold symmetry of Φ tunings is not created by biases in rat behaviour.

(**A**) Boxplots showing distributions of head direction modulation of BVCs (blue) and all other subiculum neurons (black). BVCs have low levels of head direction modulation, which is nevertheless significantly greater than that of other subiculum neurons (ANOVA Age, Cell Type: Age, $F_{(1,1589)}$=61.7, p<0.001; Cell Type, $F_{(1,1589)}$=17.6, p<0.001; Age*Cell Type, $F_{(3,1589)}$=2.14, p=0.093). (**B**) BVC head direction tuning is largely explained by positional sampling bias. Left panel: the directionally modulation of rate maps constructed under the 'distributive hypothesis' (assumption of no true head direction tuning; see Methods) is well-correlated with that of observed data. Blue line shows linear best fit, p<0.001. BVCs pooled over all age groups. Right panel: after subtraction of distributive hypothesis directional tuning from observed directional tuning, BVCs are no longer significantly more directionally tuned than the general population of subiculum neurons (ANOVA Age, Cell Type: Age, $F_{(1,1589)}$=12.8, p<0.001; Cell Type, $F_{(1,1585)}$=1.03, p=0.31; Age*Cell Type, $F_{(3,1589)}$=0.5, p=0.68). (**C**) Histograms showing preferred firing directions (PFDs) of BVCs, averaged over wall-aligned directional quadrants. Central vertical black dashed line shows wall orientations. PFDs show no significant four-fold symmetry, and are not aligned with square environment walls. Adult PFDs show a non-significant trend towards alignment with arena corners. (Rayleigh test on wrapped, quadrupled PFDs: P16-18, Z=2.5, p=0.083; P19-21, Z=2.1, p=0.122; Z=1.1, p=0.343; Z=2.8, p=0.060). n=1597 subiculum neurons.

(**D-G**) Φ tunings and four-fold symmetry in populations of simulated BVCs. (**D**) Histograms of simulated BVC Φ tunings for each age group. 50,000 BVCs were simulated using real position data, and synthetic spike trains based on Poisson distributions scaled by the BVC function, evaluated at each location. Simulated *d* and σ_0 tunings were drawn from real (age-matched) data, whereas Φ tunings were drawn from a flat distribution (see Methods for further details). The solid blue lines show the mean values across 1000 resampled populations (each with an age-matched number of BVCs), the dashed blue lines show the standard deviation. No 4-fold symmetry is observed in the simulated data. (**E**) Distribution of p-values obtained from the Rayleigh test applied to wrapped, quadrupled, Φ values shown in (D), over 1000 resampled populations. The proportions of p-values crossing the p=0.05 significance threshold were: 0.055 (P16-18), 0.043, (P19-21), 0.051 (P22-25) and 0.061 (Adults). The largest of these is not significantly different from 0.05 (Z-test for proportions, Z=1.15, p=0.28), indicating that the number of resampled populations with significant 4-fold symmetry is no greater than expected by chance. (**F**) Histograms of simulated BVC Φ tunings for each age group: simulated BVCs were created as for the populations shown in (D), with the exception that Φ tunings were drawn from real (age-matched) data. 4-fold symmetry is apparent in the simulated data, as in the real data. (**G**) Distribution of p-values obtained from the Rayleigh test applied to wrapped, quadrupled, Φ values shown in (F). Proportions of p-values crossing the p=0.05 significance threshold were: 0.968 (P16-18), 1.00, (P19-21), 0.988 (P22-25) and 1.00 (Adults).

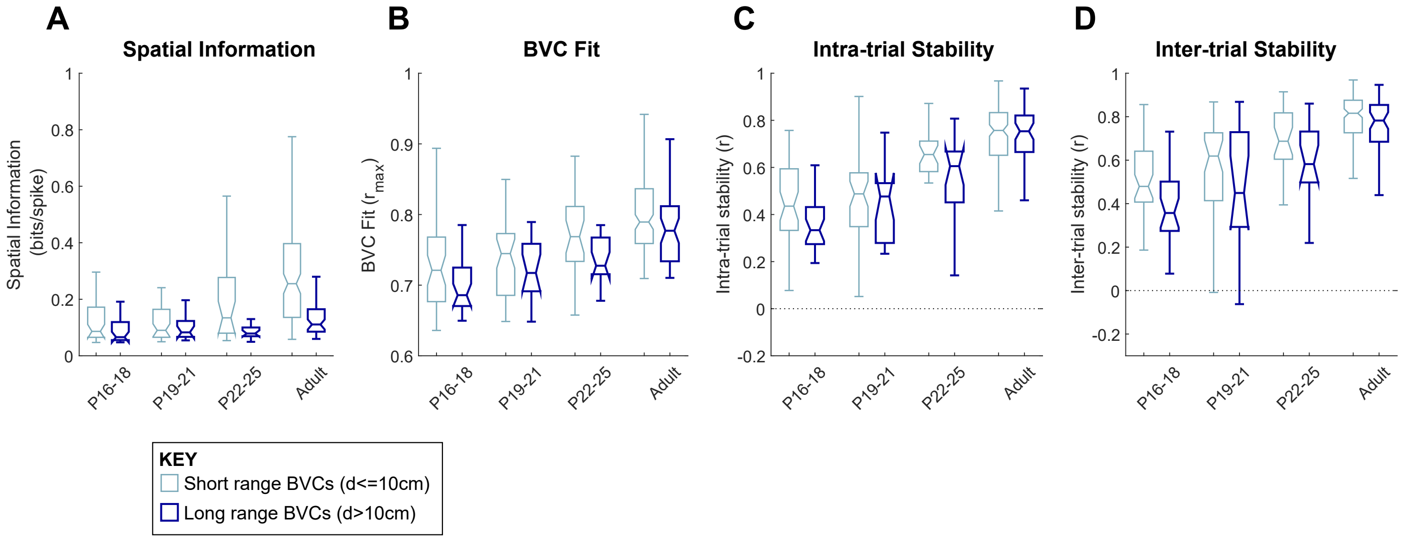

Supplementary Figure 6. Spatial firing properties of long- and short-range BVCs (*d*≤10 and *d*>10, respectively) during development. Long-range and short-range BVC do not show different maturational trends. n=368 BVCs.

(**A**) Short-range BVCs have higher spatial information than long-range BVCs; this difference significantly changes during development (ANOVA Age, *d*: Age, $F_{(3,360)}$=8.1, p=0.029; *d*, $F_{(1,360)}$=12.9, p<0.001; Age**d*, $F_{(3,360)}$=4.1, p=0.007), and is caused by an increase in the spatial information of short-range BVCs, specifically (Pairwise HSD P16 vs Adult, p<0.001). There is no significant change in the spatial information of long-range BVCs across age groups (Pairwise HSD, all comparisons to adult p>0.94).

(**B**) Short-range BVCs are better fit by the canonical BVC model than long-range BVCs; this difference does not significantly change during development (ANOVA Age, *d*: Age, $F_{(3,360)}$=36.0, p<0.001; *d*, $F_{(1,360)}$=15.8, p<0.001; Age**d*, $F_{(3,360)}$=0.43, p=0.73).

(**C**) Short-range BVCs have higher intra-trial stability than long-range BVCs; this difference does not significantly change during development (ANOVA Age, *d*: Age, $F_{(3,360)}$=90, p<0.001; *d*, $F_{(1,360)}$=4.71, p=0.031; Age**d*, $F_{(3,360)}$=1.87, p=0.135).

(**D**) Short-range BVCs are more stable between consecutive exposures to the arena than long-range BVCs, though this difference does not significantly change across development (ANOVA Age, *d*: Age, $F_{(3,360)}$=71.0, p<0.001; *d*, $F_{(1,360)}$=16.5, p<0.001; Age**d*, $F_{(3,360)}$=1.56, p=0.19). Notwithstanding the lack of a significant ANOVA interaction, there was a significant difference between long- and short-range BVC inter-trial stability for the P16-18 age bin, specifically (T-test, $t_{(47)}$=2.02, p=0.007).

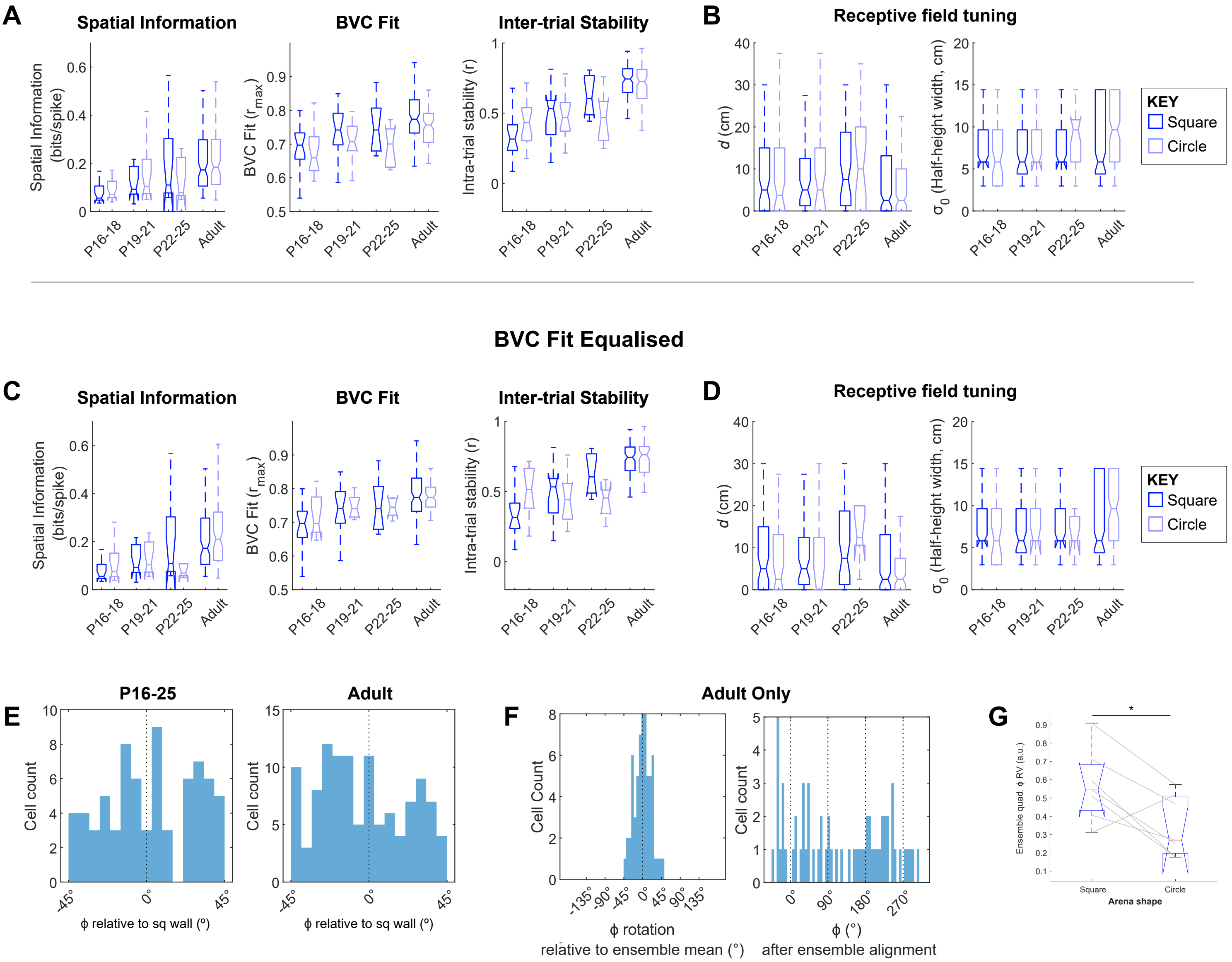

Supplementary Figure 7. Comparison of BVC spatial firing and receptive field tunings across square and circular arenas. No significant differences between square and circle were present, except for BVC model fit which was greater in the square: sub-sampling circle BVCs to equalise BVC model fit across shapes did not affect the results reported in main figure 4.

(**A**) Boxplots showing distributions of Spatial Information, BVC model fit and intra-trial stability in square and circle. BVC model fit is significantly greater in the square than the circle (Mixed ANOVA Age [across subject], Shape [within subject]: Age, $F_{(3,150)}$=17.8, p<0.001; Shape, $F_{(1,150)}$=9.34, p=0.003; Age*Shape, $F_{(3,150)}$=0.63, p=0.59), but for other measures no significant difference was observed (Spatial Information: Age, $F_{(3,150)}$=3.85, p=0.011; Shape, $F_{(1,150)}$= 0.05, p=0.82; Age*Shape, $F_{(3,150)}$= 0.18, p=0.91. Intra-trial stability: Age, $F_{(3,150)}$= 51.0, p<0.001; Shape, $F_{(1,150)}$=2.05, p=0.154; Age*Shape, $F_{(3,150)}$=5.14, p=0.002). (**B**) Boxplots showing distributions of receptive tuning properties d and σ_0: these do not significantly differ across square and circle (*d* tuning: Age, $F_{(3,150)}$=1.80, p=0.15; Shape, $F_{(1,150)}$= 1.13, p=0.29; Age*Shape, $F_{(3,150)}$=1.34, p=0.26. σ_0 tuning: Age, $F_{(3,150)}$=1.24, p=0.30; Shape, $F_{(1,150)}$=0.14, p=0.71; Age*Shape, $F_{(3,150)}$=0.49, p=0.69).

(**C**) Boxplots showing distributions of BVC spatial firing properties, following sub-sampling of circle data to equalise BVC model fits across shapes. Following sub-sampling, there was no significant difference of BVC model fit values (Independent samples ANOVA, Age, Shape: Shape, $F_{(1,225)}$=0.35, p=0.557) between square and circle, nor for spatial information (Shape, $F_{(1,225)}$=0.09, p=0.77), or intra-trial stability (Shape, $F_{(1,225)}$=0.56, p=0.45). (**D**) Boxplots showing distributions of receptive tuning properties d and σ_0, following sub-sampling of circle data to equalise BVC model fits across shapes. Following sub-sampling, there was no significant difference in either *d* ($F_{(3,225)}$=0.36, p=0.55) and σ_0 ($F_{(3,225)}$=0.18, p=0.67) across shapes.

(**E**-**G**) Equalising BVC model fit across shapes did not affect the results reported in main figure 4. (**E**) Histograms of BVC Φ tuning in the circle, averaged across 90° quadrants corresponding to walls of the square arena. The central black dashed line represents directions aligned with wall orientations. There was no significant 4-fold symmetry in the distribution of Φ tunings, either in developing rats (all developing animals grouped P16-25; Z-test on quadrupled Φ, Z=0.1, p=0.95) or in adults (Z=1.7, p=0.18). (**F**) Ensembles of ≥5 BVCs rotated coherently (left panel), and did not show 4-fold symmetry in Φ tuning even after correcting for different ensemble rotations (right panel; Z=0.3, p=0.75). Only adult data is shown, as no ensembles of ≥5 BVCs remained in development, following sub-sampling of circle data. (**G**) Rayleigh vector lengths derived from quadrupled Φ, for each ensemble with ≥5 BVCs, remain significantly greater in the square than in the circle (Wilcoxon test, p=0.031). Box plots show distribution of quad-φ RV in square and circle arenas, grey lines show change in RV for each ensemble.

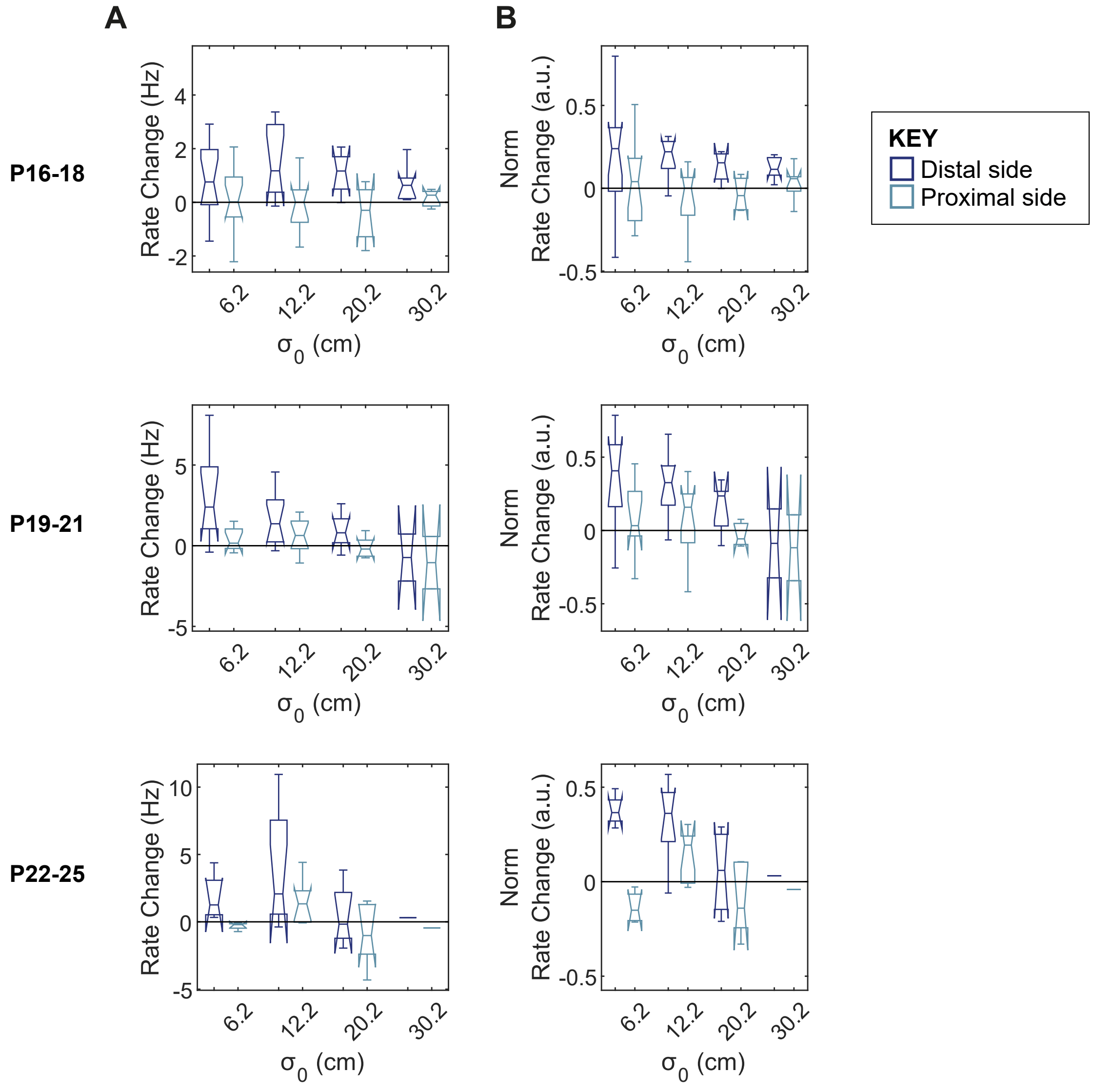

Supplemental Figure 8. BVC responses to inserted barriers in developing rats, split by σ_0 of best-fit model in the baseline trial. There is no significant inhibition of firing on the proximal side of the barrier in developing rats (ages P16-25).

(**A**) Boxplots showing distributions of absolute barrier rate change score for BVCs in each age group. Dark blue boxplots show rate changes on distal side of the barrier, light grey-blue boxplots on the proximal side of the barrier, boxplot pairs show BVC responses for each σ_0 value. There is a significant effect of σ_0 on distal side firing only at P19-21 (ANOVA, σ_0, P16-18, $F_{(3,33)}=0.64$; p=0.60; P19-21, $F_{(3,31)}=4.51$; p=0.009; P22-25, $F_{(3,18)}=0.74$; p=0.54). At P19-P21, however, no individual σ_0 groups were significantly different from zero following a sidak correction for four comparisons (corrected α=0.0127; σ_0=6.2, p=0.98; σ_0=12.2, p=0.98; σ_0=20.2, p=0.022; σ_0=30.2, p=0.060). (**B**) Boxplots showing distributions of normalised barrier rate change score for BVCs in each age group. Dark blue boxplots show rate changes on distal side of the barrier, light grey-blue boxplots on the proximal side of the barrier, boxplot pairs show BVC responses for each σ_0 value. There was no significant effect of σ_0 on distal side firing at any age (ANOVA, factor σ_0, P16-18, $F_{(3,33)}$= 1.13; p=0.35; P19-21, $F_{(3,31)}$= 1.43; p=0.25; P22-25, $F_{(3,18)}$=0.97; p=0.43).

Supplementary Figure 9

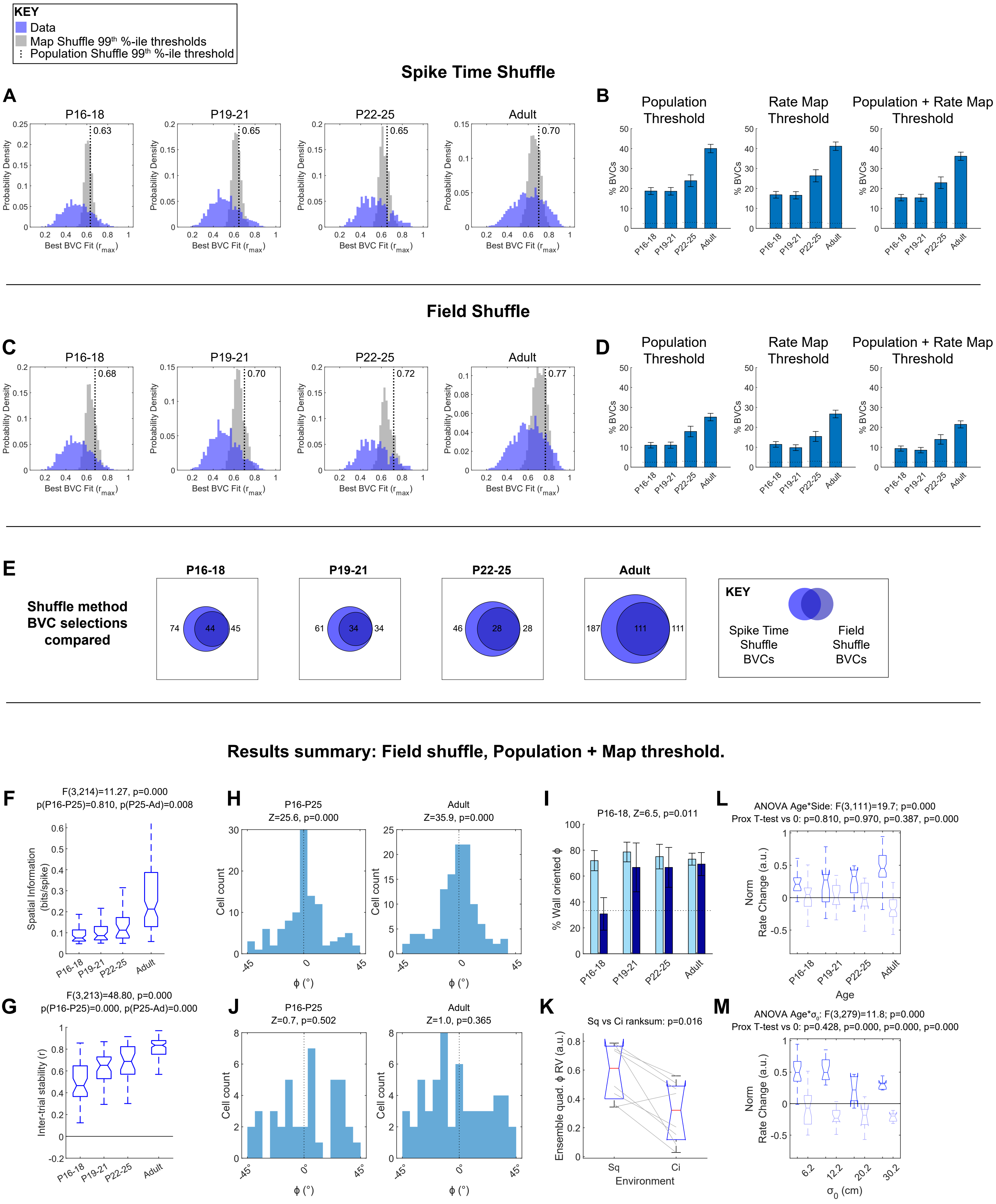

Supplementary Figure 9. Comparison of thresholding and shuffling methods for BVC definition. Key study findings are preserved when alternative, more conservative BVC classification procedures are used. n=1597 subiculum neurons.

(**A**) Histograms showing the BVC $r_{(max)}$ of real data (blue), and 99th %-ile thresholds for $r_{(max)}$ derived from shuffled data. Data was shuffled by shifting spike times relative to position (see Methods). Grey histograms show the distribution of 99th %-ile thresholds generated specifically for each cell on each trial ('rate map' threshold), the black dashed line and adjacent text shows the 99th %-ile of aggregated shuffled $r_{(max)}$ scores for all cells in an age-matched population ('population' threshold). (**B**) Percentage of cells classified as BVCs in each age group, when using only the population threshold (left), only the rate map threshold (middle) or a combination of both ($r_{(max)}$ on any trial must be greater both population and rate map thresholds; right). As expected, the rate map and population combination threshold is the most conservative (this method was used for the main manuscript).

(**C**) Histograms showing the BVC $r_{(max)}$ of real data (blue), and 99th %-ile thresholds for $r_{(max)}$ derived from shuffled data. Data was shuffled by defining spatial firing fields in the observed data rate map, and randomly rearranging within the arena ('field shuffle'; see Methods). Overall, threshold values are higher for the field shuffle than the spike time shuffle. (**D**) Percentage of cells classified as BVCs in each age group, when using only the population threshold (left), only the rate map threshold (middle) or a combination of both (left), where thresholds are derived from field-shuffled data. Substantially fewer cells are classified as BVCs, as expected from the higher thresholds.

(**E**) Venn diagrams showing the extent of overlap between spike time-shuffle and field-shuffle defined BVC populations (population + rate map threshold). Black text shows number of cells classified as BVCs using the spike-time shuffle (left), field shuffle (right) and both shuffle methods (centre). Field-shuffle defined BVCs represent a sub-set of spike-time shuffle BVCs, rather than an independent population.

(**F-H**) Summary of key study results based on BVCs defined by the most conservative threshold: field shuffle with rate map + population threshold. All key findings are preserved. (**F-G**) Spatial information (F) and inter-trial stability (G) of BVCs increase over development. Text above plots shows results of 1-way ANOVA (age), and post-hoc comparisons to adult. See main Figure 2C, E. (**H**) Histograms of BVC Φ tunings in the square environment, averaged over 90° quadrants. Black dashed line shows orientations aligned with square walls. Text above plots show results of Rayleigh test on quadrupled, wrapped Φ values. See main Figure 3E. (**I**) Proportions of BVCs with wall-aligned (light blue) or non wall-aligned (dark blue) Φ, split by long- and short-range *d* tunings. Φ tunings are not wall aligned for long-range d tunings, specifically in P16-18 pups (text above shows z-test of proportions for long-vs-short d, for P16-18). See main Figure 3H. (**J**) As for (H), but showing Φ tunings in the circular arena. See main Figure 4B. (**K**) Rayleigh vector lengths from quadrupled, wrapped Φ of ensembles of ≥5 BVCs, in both the square and the circle. Text above plot shows Wilcoxon test result for comparison of square and circle. See main Figure 4F. Note that only adult data is shown: there were no developing rat ensembles of ≥5 BVCs. (**L**) Normalised barrier scores for distal (dark blue) and proximal (light blue) sides, across development. Distal side responses increase, and proximal side responses decrease, with age. Text above plot shows results of 2-way Age*Side ANOVA, and post-hoc comparisons of distal side firing to zero. See main figure 5C. (**M**) Normalised barrier scores for distal (dark blue) and proximal (light blue) sides for adults only, split by BVC σ_0. Text above plot shows results of 2-way Age* σ_0 ANOVA, and post-hoc comparisons of distal side firing to zero. See main figure 5E.

Supplementary Figure 10

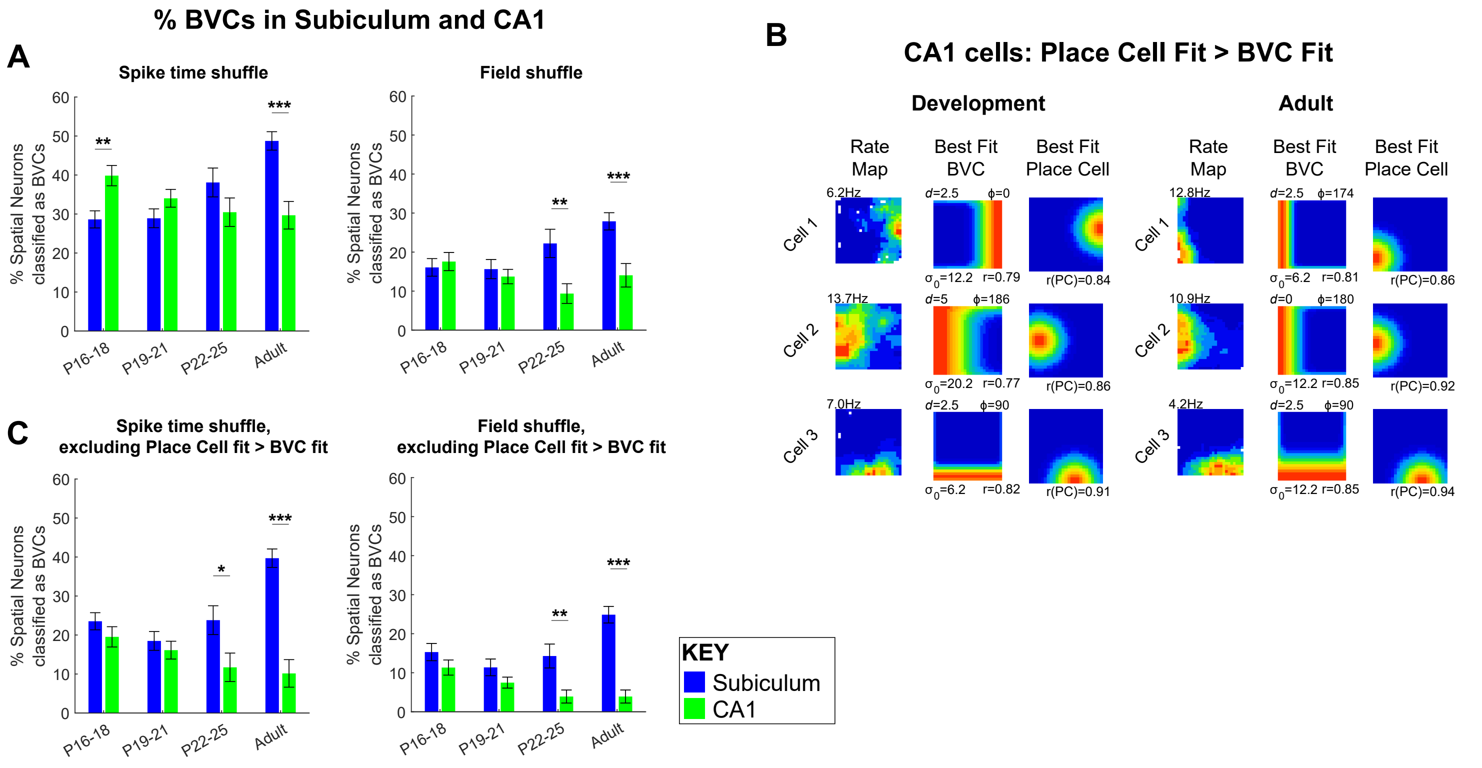

Results summary: Subiculum BVCs, Spike Time Shuffle, (Place Cell Fit>BVC Fit) cells excluded

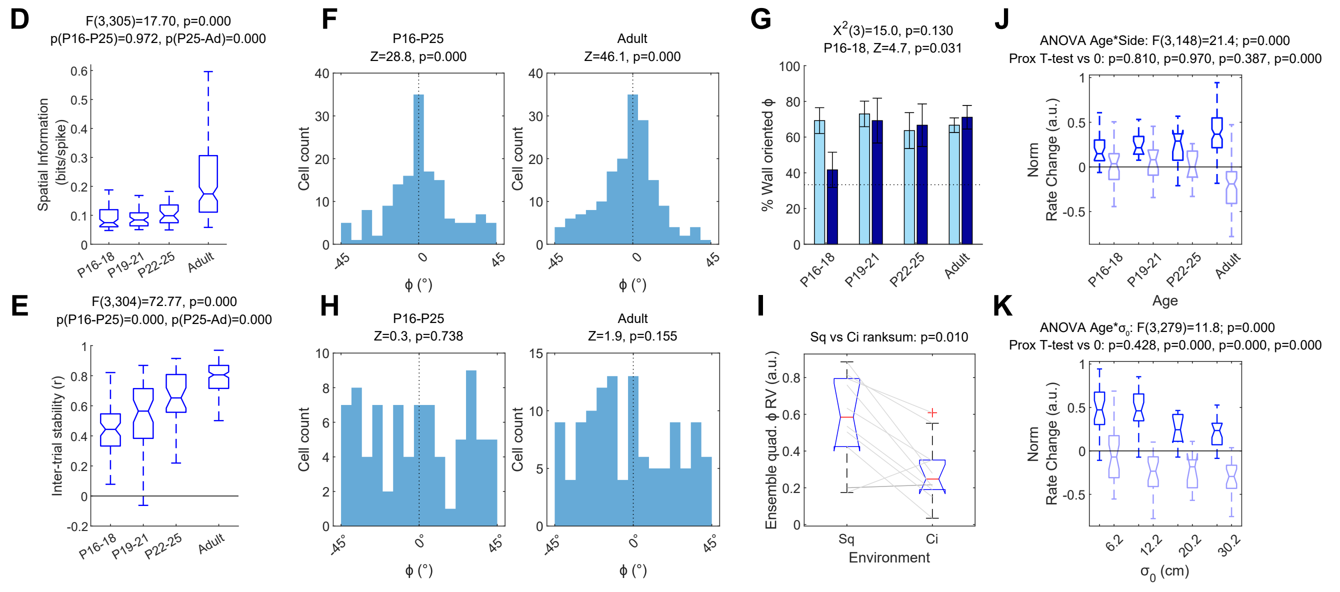

Results summary: Subiculum BVCs, Field Shuffle, (Place Cell Fit>BVC Fit) cells excluded

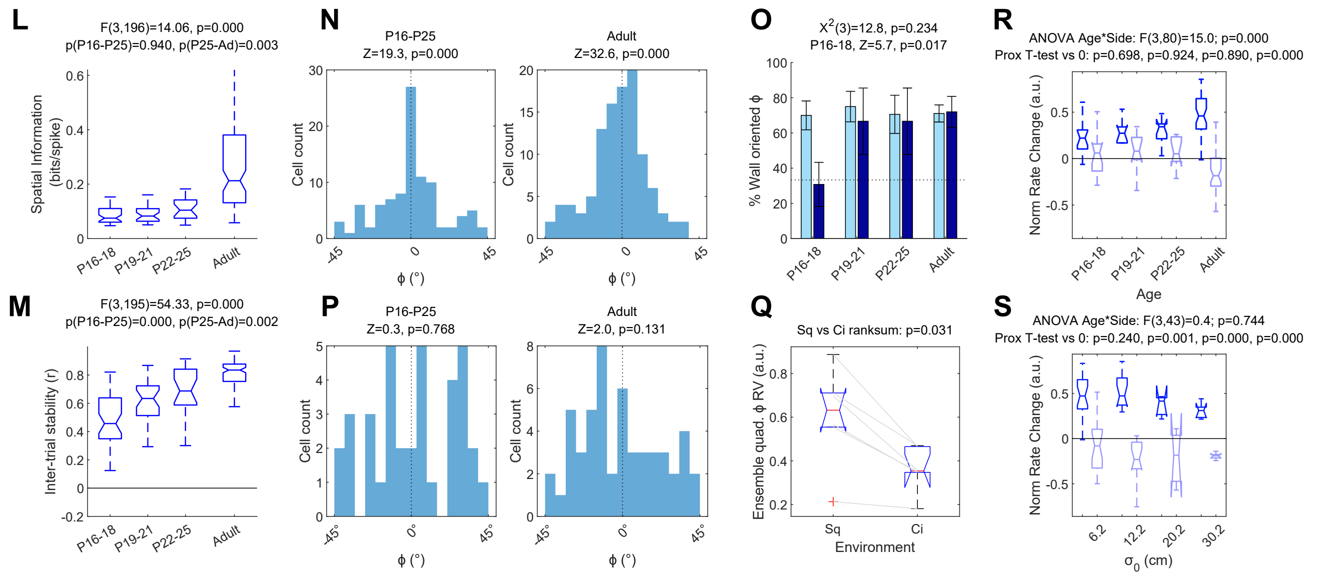

Supplementary Figure 10. Comparison of BVC detection methods across Subiculum and CA1. n=1597 subiculum neurons, 1259 CA1 neurons.

(**A**) Percentage of spatially-modulated (spatial information ≥75th %-ile shuffled population scores) neurons classified as BVCs in Subiculum and CA1, across age groups. Error bars show 95th percentile confidence interval for the proportion. Left panel shows classification using the spike-time shuffle: there is no difference in the percentage of BVCs across Subiculum and CA1 when all ages are pooled (Z-test SUB vs CA1, p=0.13), but the percentage of CA1 BVCs decreases with age, whereas the percentage of Subiculum BVCs increases (Age*Area Crosstab, $\chi^2(3)=209$, p<0.0001; Z-test SUB vs CA1, P16-18, p=0.008; Adult, p<0.001). Right panel shows classification using the field shuffle: using this method, there are significantly more BVCs in Subiculum than CA1 (Z-test SUB vs CA1, p<0.001) overall, similar numbers of BVCs across areas early in development, and significantly more BVCs in Subiculum than CA1 from weaning onwards (Age*Area Crosstab, $\chi^2(3)=208$, p<0.0001; Z-test SUB vs CA1, P22-25, p=0.004; Adult, p=0.002).

(**B**) Example CA1 cells which are significantly fit by both a BVC and a Place Cell model, but better fit by the Place Cell model. (See Methods; briefly, Place Cells were modelled as 2-dimensional Gaussians of varying positions and field widths). Left group shows examples from development (P17-P22), right group from adult animals. Right column in each group shows rate map, middle column best fitting BVC, left column best fitting place cell. Best fit $r_{(max)}$ is shown bottom right of each map. Most cells in this category displayed elongated place fields in close proximity to a boundary.

(**C**) Percentage of spatially-modulated neurons classified as BVCs in Subiculum and CA1, excluding cells better fit by the Place Cell model than the BVC model. Left panel shows classification using the spike-time shuffle: following the addition of place cell fit exclusion, there are significantly more BVCs in Subiculum than CA1 overall (Z-test SUB vs CA1, p<0.001), similar numbers of BVCs across areas early in development, and significantly more BVCs in Subiculum than CA1 from weaning onwards (Age*Area Crosstab, $\chi^2(3)=278$, p<0.0001; Z-test SUB vs CA1, P22-25, p=0.012; Adult, p<0.001). Right panel shows classification using the field shuffle: there are significantly more BVCs in Subiculum than CA1 (Z-test SUB vs CA1, p<0.001) overall, similar numbers of BVCs across areas early in development, and significantly more BVCs in Subiculum than CA1 from weaning onwards (Age*Area Crosstab, $\chi^2(3)=253$, p<0.0001; Z-test SUB vs CA1, P22-25, p=0.004; Adult, p<0.001).

(**D-K**) Key results are replicated when BVCs for which Place Cell fit is better than BVC fit were excluded from the Subiculum dataset, using the spike-time shuffle. (**D-E**) Spatial information (D) and inter-trial stability (E) of BVCs increase over development. Text above plots shows results of 1-way ANOVA (age), and post-hoc comparisons to adult. See main Figure 2C, E. (**F**) Histograms of BVC Φ tunings in the square environment, averaged over 90° quadrants. Black dashed line shows orientations aligned with square walls. Text above plots show results of Rayleigh test on quadrupled, wrapped Φ values. See main Figure 3E. (**G**) Proportions of BVCs with wall-aligned (light blue) or non wall-aligned (dark blue) Φ, split by long- and short-range *d* tunings. Φ tunings are not wall aligned for long-range d tunings, specifically in P16-18 pups (text above shows z-test of proportions for long-vs-short d, for P16-18). See main Figure 3H. (**H**) As for (F), but showing Φ tunings in the circular arena. See main Figure 4B. (**I**) Rayleigh vector lengths from quadrupled, wrapped Φ of ensembles of ≥5 BVCs, in both the square and the circle. Text above plot shows Wilcoxon test result for comparison of square and circle. See main Figure 4F. Note that only adult data is shown: there were no developing rat ensembles of ≥5 BVCs. (**J**) Normalised barrier scores for distal (dark blue) and proximal (light blue) sides, across development. Distal side responses increase, and proximal side responses decrease, with age. Text above plot shows results of 2-way Age*Side ANOVA, and post-hoc comparisons of distal side firing to zero. See main figure 5C. (**K**) Normalised barrier scores for distal (dark blue) and proximal (light blue) sides for adults only, split by BVC σ_0. Text above plot shows results of 2-way Age* σ_0 ANOVA, and post-hoc comparisons of distal side firing to zero. See main figure 5E.

(**L-S**) Key results are replicated when BVCs for which Place Cell fit is better than BVC fit were excluded from the Subiculum dataset, using the field shuffle. Format of data is as for (D-K). For (**O**) note the lack of a significant χ^2 omnibus test for changes in proportion of wall-aligned Φ, across and age and *d.* Nevertheless, there are significantly less wall-aligned Φ tunings, for long range BVCs, specifically at P16-18. For (**S**), note the lack of a significant Age*σ_0 ANOVA interaction, nevertheless, only higher σ_0 BVCs have proximal-side firing significantly less than zero, when σ_0 groups are tested

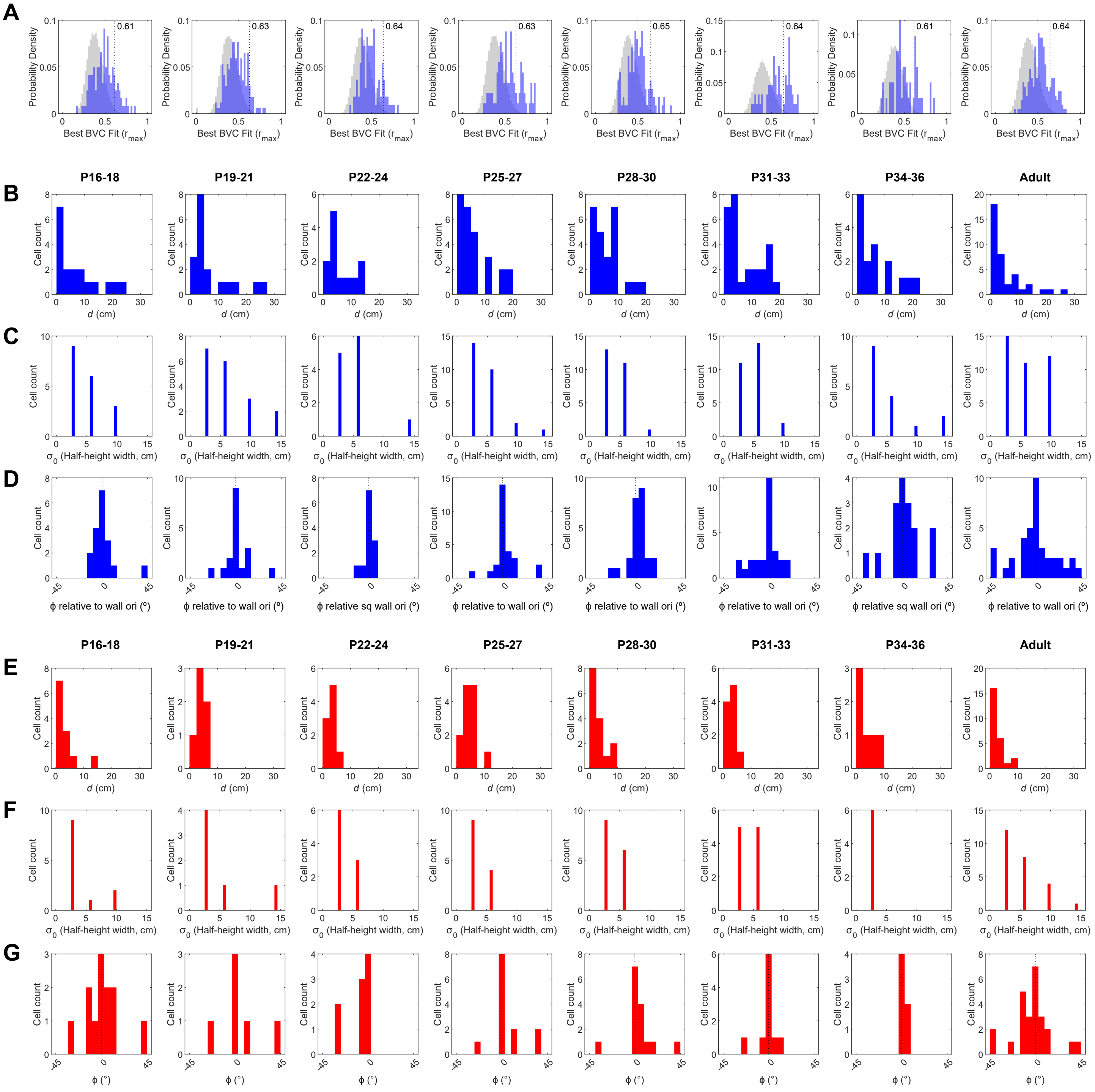

Supplemental Figure 11. Extended characterisation of BVCs and Border Cells in mEC.

(**A**) Distributions of correlations between neuronal firing rate maps and the best-fitting BVC map (r_{max}), for mEC data. Blue histograms show mEC data r_{max}, grey histograms show r_{max} based on shuffled data. Each histogram axes refers to an age group. Vertical dashed lines show the threshold r_{max} for BVC classification, defined as the 99th percentile of the spike-shifted r_{max} distributions, within each age group.

(**B**-**D**) Histograms of receptive field tuning properties for mEC BVCs in each age group. (**B**) Histograms showing all mEC BVC *d* tunings in each age group. (**C**) Counts of mEC BVC σ_0 tunings in each age group. σ_0 tunings are expressed as the half-height width of a BVC field with given σ_0, assuming *d*=0 and φ=0. (**D**) Histograms of all mEC BVC φ tunings mapped onto one 90° quadrant. 0° indicates φ tunings aligned to a wall.

(**E**-**G**) Histograms of receptive field tuning properties for mEC Border Cells in each age group. (**E**) Histograms showing all mEC Border Cell *d* tunings in each age group. (**F**) Counts of mEC Border Cell σ_0 tunings in each age group. σ_0 tunings are expressed as the half-height width of a BVC field with given σ_0, assuming *d*=0 and φ=0. (**G**) Histograms of all mEC Border Cell φ tunings mapped onto one 90° quadrant. 0° indicates φ tunings aligned to a wall. Receptive field properties of border cells were derived from the best-fitting model BVC, irrespective of whether the model BVC was determined to be an above-chance fit for the neural firing.

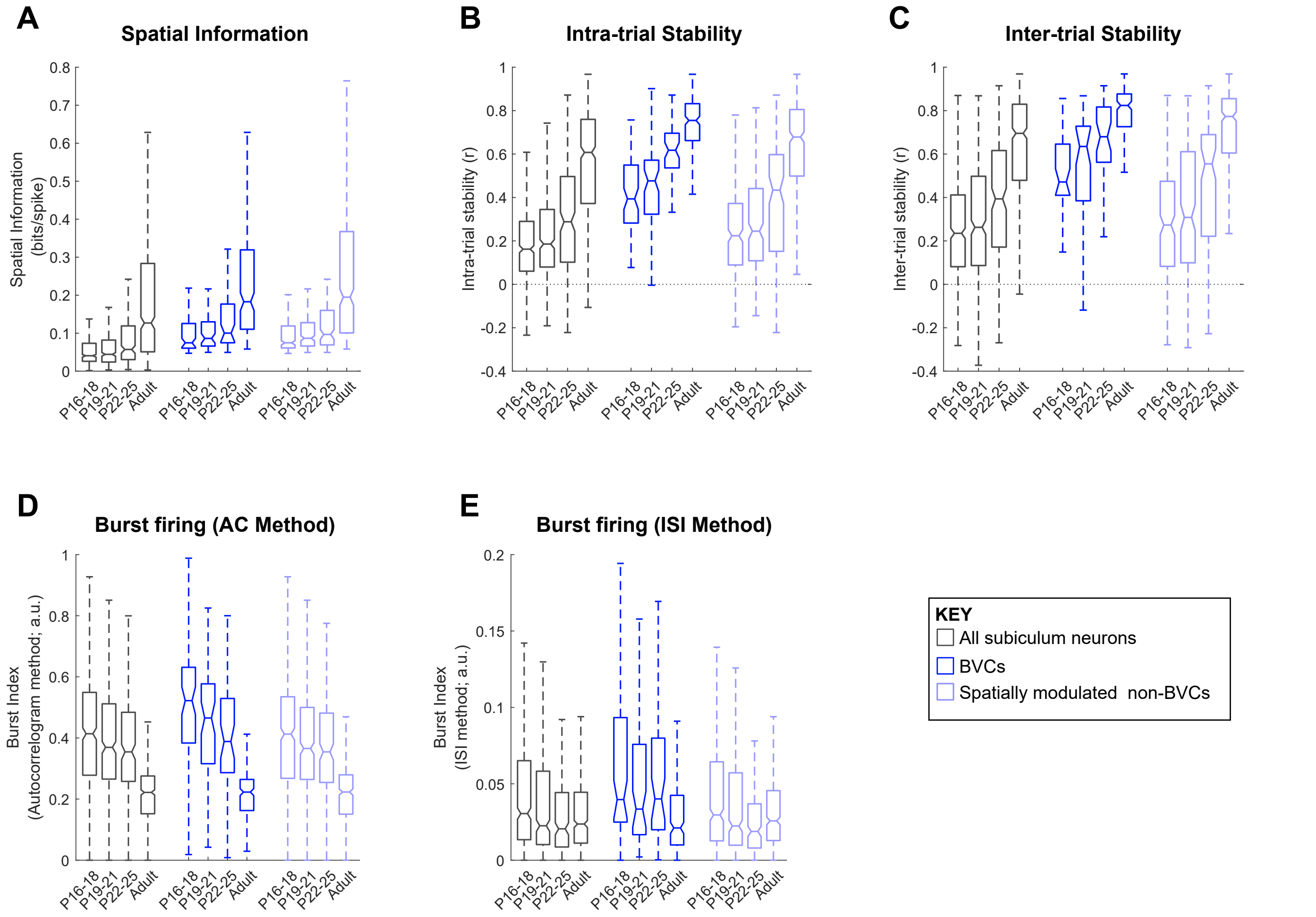

Supplementary figure 12. Spatial and burst firing properties of all subiculum neurons show a protracted development. n=1597 subiculum neurons.

(**A-C**) Boxplots of spatial firing properties for all subiculum neurons (black boxplots), along with BVCs (dark blue boxplots) and spatially-modulated non-BVCs (neurons that cross the 75th %-ile shuffle threshold for spatial information, but not the threshold for BVC model fit; light blue boxplots). (**A**) Spatial information increases over age for all neurons (ANOVA age: $F_{(1,1593)}$=89.2, p<0.001), and for spatially-modulated non-BVCs (ANOVA age:$F_{(1,599)}$=59.7, p<0.001). (For BVC statistics, see main text). (**B**) Intra-trial stability increases over age for all neurons (ANOVA age: $F_{(1,1593)}$=296, p<0.001), BVCs selectively (see main text) and spatially-modulated non-BVCs (ANOVA age: $F_{(1,599)}$=193, p<0.001). (**C**) Inter-trial stability (between consecutive baseline trials) increases over age for all neurons (ANOVA age: $F_{(1,1593)}$=215, p<0.001), BVCs selectively (see main text) and spatially-modulated non-BVCs (ANOVA age: $F_{(1,599)}$=151, p<0.001).

(**D-E**) Boxplots of burst firing properties for all subiculum neurons (black boxplots), along with BVCs (dark blue boxplots) and spatially-modulated non-BVCs (light blue boxplots).The tendency of subiculum neurons to burst fire is greater in developing rats than in adults. Bursting reduces to adult levels by either P19, or >P25, depending on the quantification method used. (**D**) Spike bursting quantified by the temporal auto-correlogram method (see Methods) significantly decreases with age, and is still immature at P25, for all subiculum neurons (ANOVA Age: $F_{(3,1593)}$=82.1, p<0.001. Post-hoc diff. to adult (Tukey): all groups p<0.001), BVCs (ANOVA Age: $F_{(1,364)}$=75.44, p<0.001. Post-hoc diff. to adult: all groups p<0.001) and spatially-modulated non-BVCs (ANOVA Age: $F_{(3,986)}$=92.38, p<0.001. Post-hoc diff. to adult: all groups p<0.001). (**E**) Spike bursting quantified by the inter-spike interval (ISI) method (see Methods) significantly changes during development, and is significantly greater from adult at P16-18, only, for all subiculum neurons (ANOVA Age: $F_{(1,1593)}$=5.79, p=0.001. Post-hoc diff. to adult: P16-18, p=0.001; P19-21, p=0.32; P22-25, p=0.99) and spatially-modulated non-BVCs (ANOVA Age: $F_{(1,599)}$=4.51, p=0.004. Post-hoc diff. to adult: P16-18, p=0.034; P19-21, p=0.911; P22-25, p=0.617). By contrast BVC bursting, as assessed by ISIs, was significantly greater than adult at all developmental time points (ANOVA Age: $F_{(1,364)}$=17.7, p<0.001. Post-hoc diff. to adult: all groups p<0.001).
