# Supplementary material for: Environment geometry alters subiculum boundary vector cell receptive fields in adulthood and early development
Source: Nat Commun. 2024 Feb 1;15:982. doi: 10.1038/s41467-024-45098-1 (PMC10834499; doi:10.1038/s41467-024-45098-1)
Supplement: Supplementary file 3 — Reporting Summary [file 41467_2024_45098_MOESM3_ESM.pdf]

nature portfolio

Corresponding author(s): Thomas J Wills

Last updated by author(s): Nov 7, 2023

Reporting Summary

Nature Portfolio wishes to improve the reproducibility of the work that we publish. This form provides structure for consistency and transparency in reporting. For further information on Nature Portfolio policies, see our Editorial Policies and the Editorial Policy Checklist.

Statistics

For all statistical analyses, confirm that the following items are present in the figure legend, table legend, main text, or Methods section.

n/a	Confirmed	
☐	☒	The exact sample size (*n*) for each experimental group/condition, given as a discrete number and unit of measurement
☐	☒	A statement on whether measurements were taken from distinct samples or whether the same sample was measured repeatedly
☐	☒	The statistical test(s) used AND whether they are one- or two-sided<br>*Only common tests should be described solely by name; describe more complex techniques in the Methods section.*
☒	☐	A description of all covariates tested
☐	☒	A description of any assumptions or corrections, such as tests of normality and adjustment for multiple comparisons
☐	☒	A full description of the statistical parameters including central tendency (e.g. means) or other basic estimates (e.g. regression coefficient) AND variation (e.g. standard deviation) or associated estimates of uncertainty (e.g. confidence intervals)
☐	☒	For null hypothesis testing, the test statistic (e.g. F, t, r) with confidence intervals, effect sizes, degrees of freedom and P value noted<br>*Give P values as exact values whenever suitable.*
☒	☐	For Bayesian analysis, information on the choice of priors and Markov chain Monte Carlo settings
☒	☐	For hierarchical and complex designs, identification of the appropriate level for tests and full reporting of outcomes
☐	☒	Estimates of effect sizes (e.g. Cohen's *d*, Pearson's *r*), indicating how they were calculated

Our web collection on statistics for biologists contains articles on many of the points above.

Software and code

Policy information about availability of computer code

Data collection	DACQ (Axona, Herts, UK) was used to acquire extracellular recording data
Data analysis	Matlab (mathworks) was used to create custom analysis routines. All custom code is available at: https://github.com/WillsCacucciLab/BVCDevPublic

For manuscripts utilizing custom algorithms or software that are central to the research but not yet described in published literature, software must be made available to editors and reviewers. We strongly encourage code deposition in a community repository (e.g. GitHub). See the Nature Portfolio guidelines for submitting code & software for further information.

Data

Policy information about availability of data

All manuscripts must include a data availability statement. This statement should provide the following information, where applicable:

- Accession codes, unique identifiers, or web links for publicly available datasets
- A description of any restrictions on data availability
- For clinical datasets or third party data, please ensure that the statement adheres to our policy

All data (Subiculum and mEC) will be stored and freely accessible to download from the UCL 'Research Data Repository' server (https://rdr.ucl.ac.uk/). The data will be linked to the DOI: 10.5522/04/24864732.

Research involving human participants, their data, or biological material

Policy information about studies with human participants or human data. See also policy information about sex, gender (identity/presentation), and sexual orientation and race, ethnicity and racism.

Reporting on sex and gender	n/a
Reporting on race, ethnicity, or other socially relevant groupings	n/a
Population characteristics	n/a
Recruitment	n/a
Ethics oversight	n/a

Note that full information on the approval of the study protocol must also be provided in the manuscript.

Field-specific reporting

Please select the one below that is the best fit for your research. If you are not sure, read the appropriate sections before making your selection.

☒ Life sciences ☐ Behavioural & social sciences ☐ Ecological, evolutionary & environmental sciences

For a reference copy of the document with all sections, see nature.com/documents/nr-reporting-summary-flat.pdf

Life sciences study design

All studies must disclose on these points even when the disclosure is negative.

Sample size	Sample sized was based on those required in previous studies to reliably estimate developmental trends in neuronal firing (e.g. Wills, Cacucci et al., 2010, Science; Bjerknes et al., 2014, Neuron).
Data exclusions	Data were excluded only on the basis of insufficient sampling of neuronal spiking (minimum rate 0.2Hz) or insufficient positional sampling (<80% of environment covered). These exclusions were in place to prevent insufficiently well-sampled data leading to spurious behavioural correlates of neuronal activity.
Replication	No replication was performed: due to the difficulty of collecting in vivo neuronal recordings during development, it was not feasible to collect additional datasets for replication.
Randomization	Randomization was not relevant to our study, as data was primarily collected within subject. All developing rats could potentially contribute to all age groups (though not all did, due to tetrodes not being well-positioned to record cells on some days). The majority of neurons studied were exposed to all experimental conditions (different shapes, barriers).
Blinding	Experimenters were not blinded to age. This is not possible as age can be discerned from animal size and behaviour, when experiments are run.

Reporting for specific materials, systems and methods

We require information from authors about some types of materials, experimental systems and methods used in many studies. Here, indicate whether each material, system or method listed is relevant to your study. If you are not sure if a list item applies to your research, read the appropriate section before selecting a response.

Materials & experimental systems

n/a	Involved in the study	
☒	☐	Antibodies
☒	☐	Eukaryotic cell lines
☒	☐	Palaeontology and archaeology
☐	☒	Animals and other organisms
☒	☐	Clinical data
☒	☐	Dual use research of concern
☒	☐	Plants

Methods

n/a	Involved in the study	
☒	☐	ChIP-seq
☒	☐	Flow cytometry
☒	☐	MRI-based neuroimaging

April 2023

Animals and other research organisms

Policy information about studies involving animals; ARRIVE guidelines recommended for reporting animal research, and Sex and Gender in Research

Laboratory animals	Rattus norvegicus, Lister Hooded strain. Developing animals were post-natal day 16-25, adult animals were aged 6-9 months.
Wild animals	no wild animals were used.
Reporting on sex	Only male subjects were used.
Field-collected samples	No field samples were used
Ethics oversight	Work was conducted in compliance with ASPA (1986) UK legislation. The PPL numbers were 70/8636 and 70/7136. The experiments were approved by the UCL ethical review panel.

Note that full information on the approval of the study protocol must also be provided in the manuscript.

Plants

Seed stocks	n/a
Novel plant genotypes	n/a
Authentication	n/a
